# Molecular Pharmacology and Pathology of Strokes

**DOI:** 10.3390/ijms19124103

**Published:** 2018-12-18

**Authors:** Joen-Rong Sheu

**Affiliations:** Graduate Institute of Medical Sciences, College of Medicine, Taipei Medical University, 250 Wu-Hsing Street, Taipei 110, Taiwan; sheujr@tmu.edu.tw; Tel.: +886-2-2736-1661 (ext. 3199); Fax: +886-2-2739-0450

Stroke, an important neurological disease, is becoming an increasingly non-communicable ailment and is the second leading cause of death after coronary heart disease in developed countries [1]. Present treatment options for stroke are adapting lifestyle practice, diabetes treatment, drugs, and other factors management, but no cure is yet available, despite new insights into the molecular and therapeutic targets. Discoveries in explicating the molecular pharmacology in cerebrovascular function and thrombosis have led to significant advancements in the current treatment paradigm for patients with stroke. Hence, this Special Issue invited scientific papers and reviews from researchers to provide solid evidence from a molecular point of view to scrutinize the molecular pharmacology and pathology of strokes. Platelet activation plays a major role in cardio and cerebrovascular diseases. Platelets also play a key role in the hemostatic process and are associated with various pathological events, such as arterial thrombosis and atherosclerosis. While currently used anti-platelet drugs such as aspirin and clopidogrel demonstrate efficacy in many patients, they exert undesirable side effects. Therefore, the development of effective therapeutic strategies for the prevention and treatment of thrombotic diseases is a demanding priority. Recently, precious metal drugs have conquered the subject of metal-based drugs, and several investigators have moved their attention to the synthesis of various ruthenium (Ru) and iridium (Ir) complexes due to their prospective therapeutic values.

In this Special Issue, the authors Hsia et al. [2] found that Ir (III)-derived complex, (Ir-11), showed potent antiplatelet activity by inhibiting platelet activation through the suppression of the phosphorylation of phospholipase Cγ2 (PLCγ2), protein kinase C (PKC) cascade and the subsequent suppression of Akt and mitogen-activated protein kinases (MAPKs) activation, ultimately inhibiting platelet aggregation. A detailed in vitro antiplatelet, in vivo antithrombotic and structure-activity relationship (SAR) study was performed on newly synthesized Ir complexes, Ir-1, Ir-2 and Ir-4, in agonists-induced human platelets [3]. This study found that Ir-1 expressively suppressed collagen-induced Akt, PKC, p38MAPKs and JNK phosphorylation. Interestingly, platelet function analyzer (PFA-100) showed that Ir-1 caused a significant increase in collagen-adenosine diphosphate (C-ADP) induced closure times in mice, but Ir-2 and 4 had no effect on these reactions. Moreover, Ir-1 significantly prolonged the platelet plug formation, increased tail bleeding times and reduced the mortality of adenosine diphosphate (ADP)-induced acute pulmonary thromboembolism in mice. Ir-1 has no substitution on its phenyl group; a water molecule (like cisplatin) can replace its chloride ion and, hence, the rate of hydrolysis might be tuned by the substituent on the ligand system. These features might have played a role for the observed effects of Ir-1. These results indicate that Ir compounds may be a lead compound to design new antiplatelet drugs for the treatment of thromboembolic diseases.

A major review has summarized the antiplatelet activity of newly synthesized ruthenium (Ru)-based compounds (TQ-1, 2, 3, 5 and 6) with their potential molecular mechanisms [4]. This paper condenses the antiplatelet activity of Ru compounds with the major aspects of (i) ruthenium compounds on adenosine triphosphate (ATP) and [Ca^2+^]i mobilization in antiplatelet therapy, (ii) ruthenium compounds on MAPKs in antiplatelet effects, (iii) ruthenium compounds on cyclic adenosine 3′,5′-monophosphate (cAMP) and cyclic guanosine 3′,5′-cyclic monophosphate (cGMP) signaling in platelets, (iv) molecular targets of ruthenium compounds in antiplatelet property, (v) antithrombotic effect of ruthenium compounds and (vi) safety and toxicity of ruthenium compounds in platelets. The given information in platelet biology and the functions of ruthenium compounds used for antiplatelet therapy will provide new opportunities to develop therapeutic strategies aimed at promoting cerebro/cardiovascular health.

In addition to the metal complexes, a natural compound, licochalcone A (LA), an active ingredient of licorice, has been studied for its antiplatelet effects. This study demonstrated that LA effectively reduced platelet activation and thrombus formation through the inhibition of PLCγ2-PKC, Akt, and MAPK pathways, without the side effect of bleeding [5]. These results concluded that LA may provide a safe and alternative therapeutic approach for preventing thromboembolic disorders such as stroke.

In the paper by Janicki et al. [6], the population-specific associations of deleterious rare variants in coding region of P2RY1-P2RY12 purinergic receptor genes in large-vessel ischemic stroke patients were studied. In this paper, the authors identified the association between ischemic stroke (IS) and six rare functional and damaging variants in the purinergic genes (P2RY1 and P2RY12 locus). The predicted properties of the most damaging rare variants in P2RY1 and P2RY12 were confirmed by using mouse fibroblast cell cultures transfected with plasmid constructs containing cDNA of mutated variants (FLIPR on FlexStation3). This study recognized a reputed role for rare variants in P2RY1 and P2RY12 genes involved in platelet reactivity on large-vessel IS susceptibility in a Polish population.

Traumatic brain injury (TBI) is one of the leading causes of mortality worldwide and leads to persistent cognitive, sensory, motor dysfunction, and emotional disorders. Yen et al. [7] discovered the neuroprotective effects of platonin, a cyanine photosensitizing dye, against TBI in a controlled cortical impact (CCI) injury model in mice. They found that platonin reduced the neurological severity score, general locomotor activity, and anxiety-related behavior, and improved the rotarod performance of CCI-injured mice, and it reduced lesion volumes, the expression of cleaved caspase-3, and microglial activation in TBI-insulted brains. This natural compound also suppressed mRNA expression of caspase-3, caspase-1, cyclooxygenase-2 (COX-2), tumor necrosis factor-α (TNF-α), interleukin-6 (IL-6), and interleukin-1β (IL-1 β). This study suggested that treatment with platonin exhibited prominent neuroprotective properties against TBI in a CCI mouse model through its anti-inflammatory, anti-apoptotic, and anti-free radical capabilities, and this data indicates that platonin may be a potential therapeutic medicine for use with TBI. 

Protocatechuic acid (PCA), a major metabolite of the antioxidant polyphenols, has been found in green tea. Protocatechuic acid had been reported as having antioxidant effects in healthy cells and anti-proliferative effects in tumor cells. Global cerebral ischemia (GCI) is one of the main roots of hippocampal neuronal death. Ischemic damage can be saved by early blood reperfusion. Nevertheless, under some situations reperfusion can activate a cell death process started by the reintroduction of blood, followed by the production of superoxide, a blood brain barrier (BBB) disruption and microglial activation. In the present Special Issue, Kho et al. [8] found that PCA significantly diminishes degenerating neuronal cell death, oxidative stress, microglial activation, astrocyte activation and BBB disruption. Moreover, an ischemia-induced reduction in glutathione concentration in hippocampal neurons is recovered by PCA administration. The obtained results provide evidence supporting the idea that administration of PCA may act as a promising tool for decreasing hippocampal neuronal death after global cerebral ischemia. 

The importance of task-specific training (TST) as a neuromotor intervention in neurological restoration has been evidenced [9]. Task-specific training can improve experience-dependent motor skill learning and neural plastic changes in animal and human brains [9]. The effectiveness of TST alone and combined with DNA methyltransferase inhibitor in chronic stroke recovery was investigated. The authors found TST and TST with DNA methyltransferase inhibitor significantly increased the crossing fibers from the contralesional red nucleus, reticular formation in medullar oblongata, and dorsolateral spinal cord. Functional recovery after chronic stroke may involve axonal plasticity and increased mature brain-derived neurotrophic factor (BDNF). These results suggest that combined therapy to enhance axonal plasticity based on TST and DNA methyltransferase inhibitor constitutes a promising approach for promoting the recovery of function in the chronic stage of stroke [10].

Ischemic stroke can cause enhanced frailty. Numerous studies have been proposed in laboratory animals and patients to reduce frailty and subsequent risk of stroke and cognitive decline. Whole body vibration (WBV) improves cerebral function and cognitive ability that deteriorates with increased frailty. A study examined in this Special Issue to test the efficacy of WBV in reducing post-ischemic stroke frailty and brain damage in reproductively senescent female rats. The results establish a noteworthy reduction in inflammatory markers and infarct volume with substantial increases in brain-derived neurotrophic factor and improvement in functional activity after transient middle cerebral artery occlusion (tMCAO) in middle-aged female rats that had been treated with WBV as compared to the no-WBV group. The conclusion of this study may simplify a faster translation of the WBV involvement for improved outcome after stroke, principally among frail women [11].

Overall, we anticipate that readers will find that this Special Issue reports the significant challenges, predictions, and present advances that are currently being faced by stroke research, with the possibility of inspiring the application of novel drug development for enriching the devotion and treatment of patients with cardiovascular diseases. 
